# Tanshinone IIA prevents platelet activation and down-regulates CD36 and MKK4/JNK2 signaling pathway

**DOI:** 10.1186/s12872-019-01289-z

**Published:** 2020-02-14

**Authors:** Hua Wang, Lin Zhong, Shaohua Mi, Nianpeng Song, Wei Zhang, Ming Zhong

**Affiliations:** 1 The Key Laboratory of Cardiovascular Remodeling and Function Research, Chinese Ministry of Education, Chinese National Health Commission and Chinese Academy of Medical Sciences, The State and Shandong Province Joint Key Laboratory of Translational Cardiovascular Medicine, Department of Cardiology, Qilu Hospital of Shandong University, No 107 Wenhua West Road, Ji’nan, 250012 China; 2 Department of Cardiology, Yantai Yuhuangding Hospital, Qingdao Medical College, Qingdao University, Yantai, China

**Keywords:** Tanshinone IIA, Platelet activaton, CD36, Atherothrombosis

## Abstract

**Background:**

Tanshinone IIA (TS IIA), a multi-pharmaceutical compound from traditional Chinese herb, is effective for treatment of atherothrombosis. However, the underlying mechanisms of TS IIA-mediated anti-platelet activation effect are still poorly understood. As shown in our previous study, platelet-derived microvesicles (PMVs) generated in response to oxidant insult could activate CD36/mitogen-activated protein kinase kinase 4/Jun N-terminal kinase 2 (CD36/MKK4/JNK2) signals and lead to platelet activation. The present study aims to investigate the effect of TS IIA on platelet activation and the possible mechanisms.

**Methods:**

The production of PMVs induced by Interleukin 6 (IL-6) was detected by flow cytometry. We performed activating studies of platelets with PMVs derived from IL-6–treated platelets (IL-6–PMVs) in vitro. Sometimes, platelet suspensions were incubated with serial concentrations of TS IIA for 15 min before being stimulated with IL-6–PMVs. Expression of platelet integrin α_IIb_β_3_ and CD36 was detected by flow cytometry. Phosphorylation of MKK4 and JNK were detected by immunoblotting.

**Results:**

Here we demonstrated firstly that TS IIA could prevent platelet activation induced by PMVs and down-regulates CD36 and MKK4/JNK2 signaling pathway. CD36 may be the target of atherosclerosis (AS)-related thrombosis.

**Conclusions:**

This study showed the possible mechanisms of TS IIA-mediated anti-platelet activation and may provide a new strategy for the treatment of AS-related thrombosis by targeting platelet CD36.

## Background

Atherothrombosis is the leading cause of morbidity and mortality worldwide. Tanshinone IIA (TS IIA), a pharmacological active components extracting from the rhizome of the Chinese herb Salvia miltiorrhiza Bunge, has been used widely for effective treatment of atherothrombosis in traditional Chinese medicine for a long history [1, 2]. However, its underlying molecular mechanisms of anti-thrombotic effect are still poorly understood. Platelet activation has been implicated as central components of the Virchow’s triad for thrombus formation [3]. Platelet-derived microvesicles (PMVs) have a critical role in activation of platelets [4]. It is well established that PMVs, carrying inflammation factors, oxidation factors, various growth factors and coagulation factors, can participate in a wide range of pathophysiological process [5, 6]. Similarly, TS IIA has been reported to have multi-pharmaceutical activities, such as anti-inflammatory capacity [7], antioxidant properties [8] and the ability to prevent platelet activation [9]. Moreover, Tang et al. [10] had showed that TS IIA attenuates atherosclerosis in ApoE (-/-) mice through down-regulation of CD36 expression. CD36, a class B scavenger receptor, promotes platelet activation by binding with PMVs. As shown in our previous study, PMV-CD36 complex could activate mitogen-activated protein kinase kinase 4/Jun N-terminal kinase 2 (MKK4/JNK2) signals and lead to platelet activation [11]. We hypothesized that TS IIA could inhibit platelet activation by suppressing the effects of MKK4/JNK2 signaling pathway mediated by PMV-CD36 complex, which has never been reported.

The present study intends to investigate whether TS IIA could prevent platelet activation induced by PMV-complex and down-regulates MKK4/JNK2 signaling pathway. PMV-CD36 complex could be the target of atherosclerosis (AS)-related thrombosis.

## Methods

### Reagents

TS IIA was obtained commercially (Xi’an Honson Biotechnology, China). IL-6 (0203B16) was from PEPROTECH, USA*Inhibitory reagents.* SP600125 was from Calbiochem (San Diego, CA, USA).

#### Reagents used for flow cytometry

Phycoerythrin (PE)- cy™5-conjugated mouse anti-CD41a antibody (clone HIP8), fluorescein isothiocyanate (FITC)-conjugated PAC-1, PE-conjugated anti-CD36 (clone CB38) and PE-conjugated CD36 isotype control (clone G155-228) were purchased from BD Biosciences/Pharmingen (San Jose, CA, USA).

#### Reagents used for western-blot analyses

Monoclonal rabbit anti-MKK4, phospho-MKK4 (p- MKK4), c-Jun NH2-terminal kinase (JNK) and mouse anti–phospho-JNK (p-JNK) were from Cell Signaling Technology (Danvers, MA, USA).

### Platelet isolation

Blood from healthy volunteers was collected into sodium citrate anticoagulant. Donors had not taken aspirin or NSAIDs for 2 weeks [11, 12]. At room temperature, platelet-rich plasma (PRP) was separated by centrifugation (10 min at 120 g). Platelets were obtained from PRP after centrifugation and washed 3-times with equal volumes of modified Tyrode’s Buffer, composed of 137 mmol/L NaCl, 2.7 mmol/L KCl, 12 mmol/L NaHCO3, 0.4 mmol/L NaH2PO4, 5 mmol/L HEPES, 0.1% glucose and 0.35% bovin serum albumin (BSA), 100 nmol/L PG-E1 (Sigma-Aldrich), pH 7.2. Platelet suspension was adjusted to 1 × 10^6^/mL. The final platelet was resuspended at a concentration of 1 × 10^6^ platelets/mL in modified Tyrode’s buffer.

### Isolation of PMVs derived from platelet supernatant

Platelet suspension (1 × 10^6^/mL) was exposed to buffer control, 1 μg/mL IL-6 (Pepro Tech, USA) or 10 mol/L adenosine diphosphate (ADP) for 60 min at 37 °C. Culture supernatants were collected, and nonviable cells and cell fragments were removed by centrifugation at 3000 g for 10 min. Subsequently, the supernatants were then sedimented at 15,000 g for 60 min at 4 °C to pellet PMVs. Then the PMV pellets were washed twice to avoid contamination of IL-6 and resuspended in modified Tyrode buffer. The PMVs were characterized by flow cytometry (BD Biosciences) [11, 13] and the protein content was measured by the Bradford Protein Assay Kit (Beyotime, Jiangsu, China).

### Flow cytometric measurement of platelet integrin α_IIb_β_3_ (PAC-1) and CD36 expression

Washed platelet suspensions (1 × 10^6^/mL) were incubated with IL-6–PMVs (MVs derived from IL-6 stimulated platelets). In some studies, to elucidate the effect of TS IIA on platelet activation or platelet CD36 expression, resting platelets were treated independently with serial concentrations of TS IIA for 15 min before exposure to PMVs. Expression of platelet integrin α_IIb_β_3_ and CD36 was analyzed by flow cytometry. Activated platelet integrin α_IIb_β_3_ were labeled by adding 5 μL PEcy5-conjugated anti-CD41a antibody and 5 μL FITC-conjugated PAC-1 antibody. 5 μL PEcy5-conjugated anti-CD41a antibody and 5 μL PE-conjugated anti-CD36 antibody were used to detect platelet CD36 expression in the dark for 15 min. MKK4/JNK2 mediated signaling pathways were blocked using JNK inhibitor SP600125.

### Western blot analysis

Protein sample preparation and western blot analysis were performed as described in previous studies [11] with primary antibodies against p-MKK4 or total MKK4 and p-JNK or total JNK (Cell Signaling Technology, Danvers, MA), followed by anti-IgG horseradish peroxidase–conjugated secondary antibody.

### Statistical analysis

Statistical analyses were carried out using SPSS 18.0 (SPSS Inc., Chicago, IL). Data are presented as means ± SD. Comparison among groups involved an ANOVA with post-hoc least-significant differences *t* test (for continuous data). A *P* value less than 0.05 was considered statistically significant.

## Results

### IL-6 stimulation increases the formation of PMVs

**Fig. 1:**
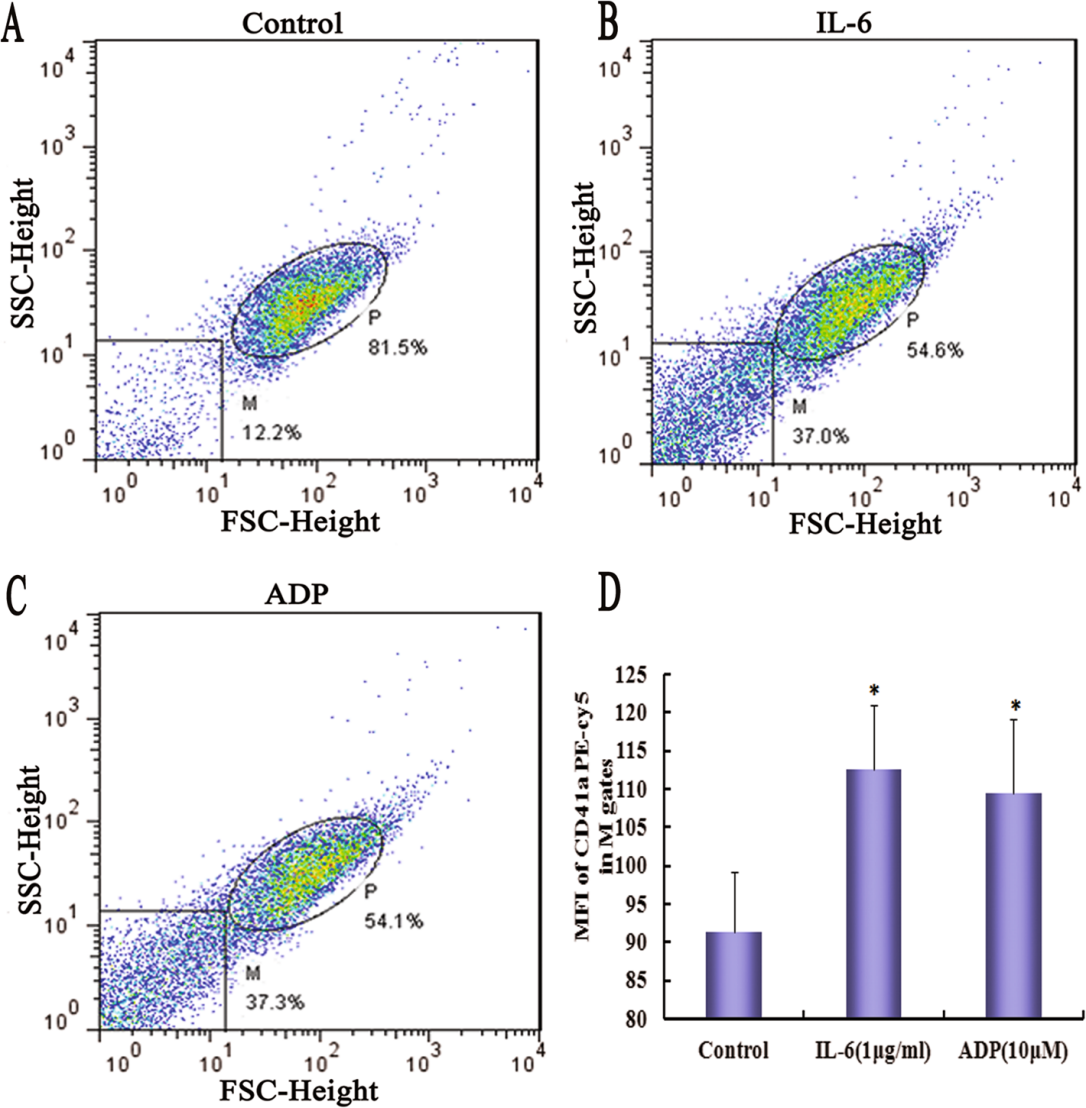
Flow cytometric analysis of platelet-derived microvesicles (PMVs). Resting platelet suspensions (1 × 10^6^/mL) were treated with buffer control **a**, 1 μg/mL Interleukin 6 (IL-6) **b** or 10 μmol/L adenosine diphosphate (ADP) **c** for 60 min at 37 °C. Representative flow cytometric scatter plot (M in Fig. 1a–c). P and M indicate the gates for platelets and PMVs, respectively. **d** Mean fluorescent intensity (MFI) of CD41a PEcy5 in M gates. Data are means ± SD from 3 separate experiments. * *P <* 0.05 compared with control

Flow cytometric analysis of washed platelets showed an increase in the formation of PMVs after treatment with 1 μg/mL IL-6 or 10 μmol/L traditional agonist ADP (Fig. 1). The flow cytometric scatter patterns indicate the formation of PMVs in the lower lefthand gate of the scatter plot (M gates in Fig. 1a–c). The PMVs were identified by labeling with PEcy5-conjugated anti-CD41a antibody. Compared with untreated platelets, the mean fluorescence intensity (MFI) of CD41a in M gates increased significantly in the IL-6 or ADP treated platelets (Fig. 1d).

### IL-6–PMVs induced platelet activation in a CD36-dependent way

**Fig. 2:**
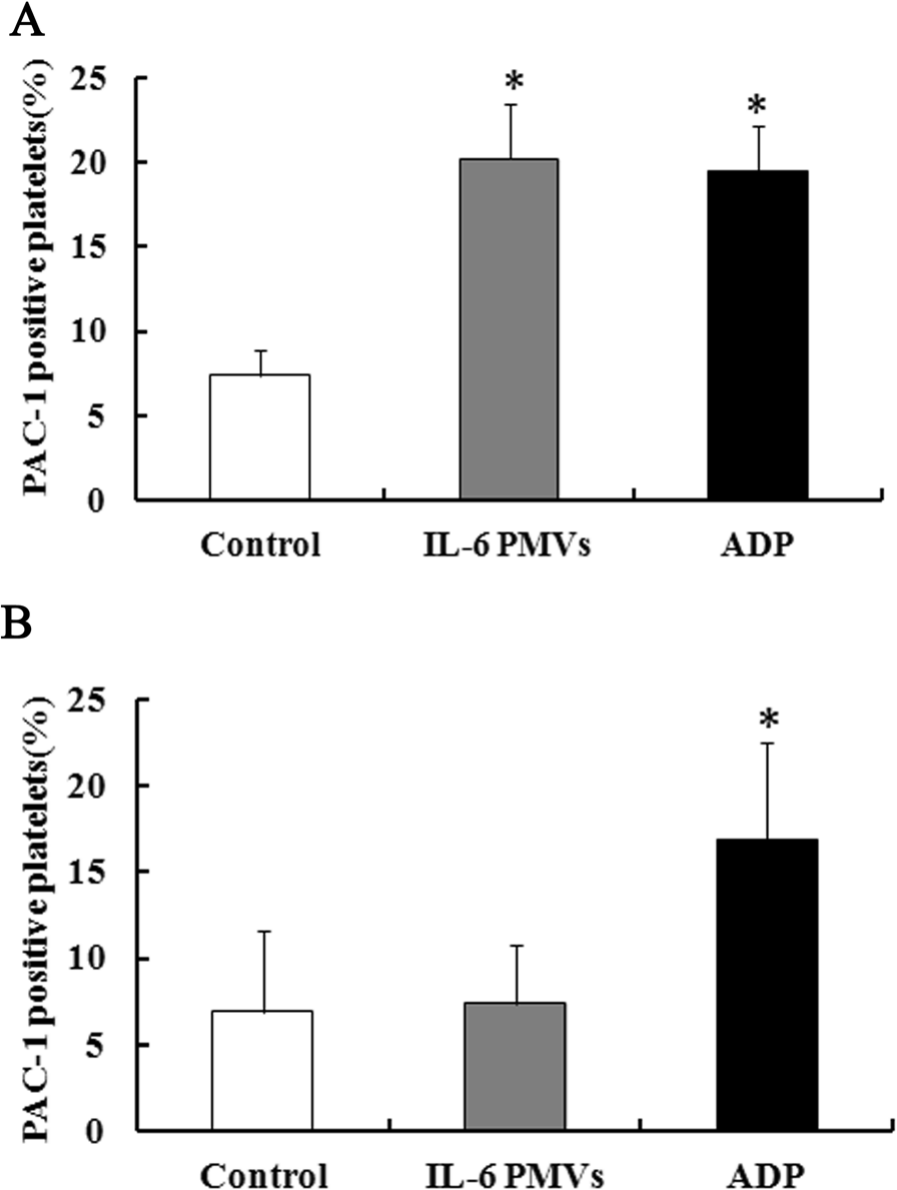
IL-6–PMVs activate platelets in a CD36-dependent manner. Percentage of PAC-1 positive platelets, after incubation of CD36-positive platelets **a** or CD36-deficient platelets **b** with IL-6–PMVs for 30 min. Data are means ± SD from 3 separate experiments. * *P <* 0.05 compared with untreated control

It is reported that 3–4% of Asian are platelet CD36-deficient subjects [14]. We found 2 CD36-deficient volunteers. The CD36-deficient platelets, which were identified by flow cytometry, unable to bind PE-conjugated anti-CD36 antibody [11]. We incubated CD36-positive and CD36-deficient platelets with IL-6–PMVs (30 μg/mL) for 30 min respectively. Platelet activation is recognized as a conformation change in integrin αIIbβ3. The percentage of PAC-1 (recognizing the activated platelet integrin αIIbβ3) of CD36-positive platelets increased significantly stimulated by IL-6–PMVs, with similar effect as 10 μmol/L ADP (Fig. 2a). CD36 deficiency in platelets prevent the effect of IL-6–PMVs while CD36-deficient platelets respond normally to ADP (Fig. 2b).

### TS IIA blocks platelet CD36

**Fig. 3:**
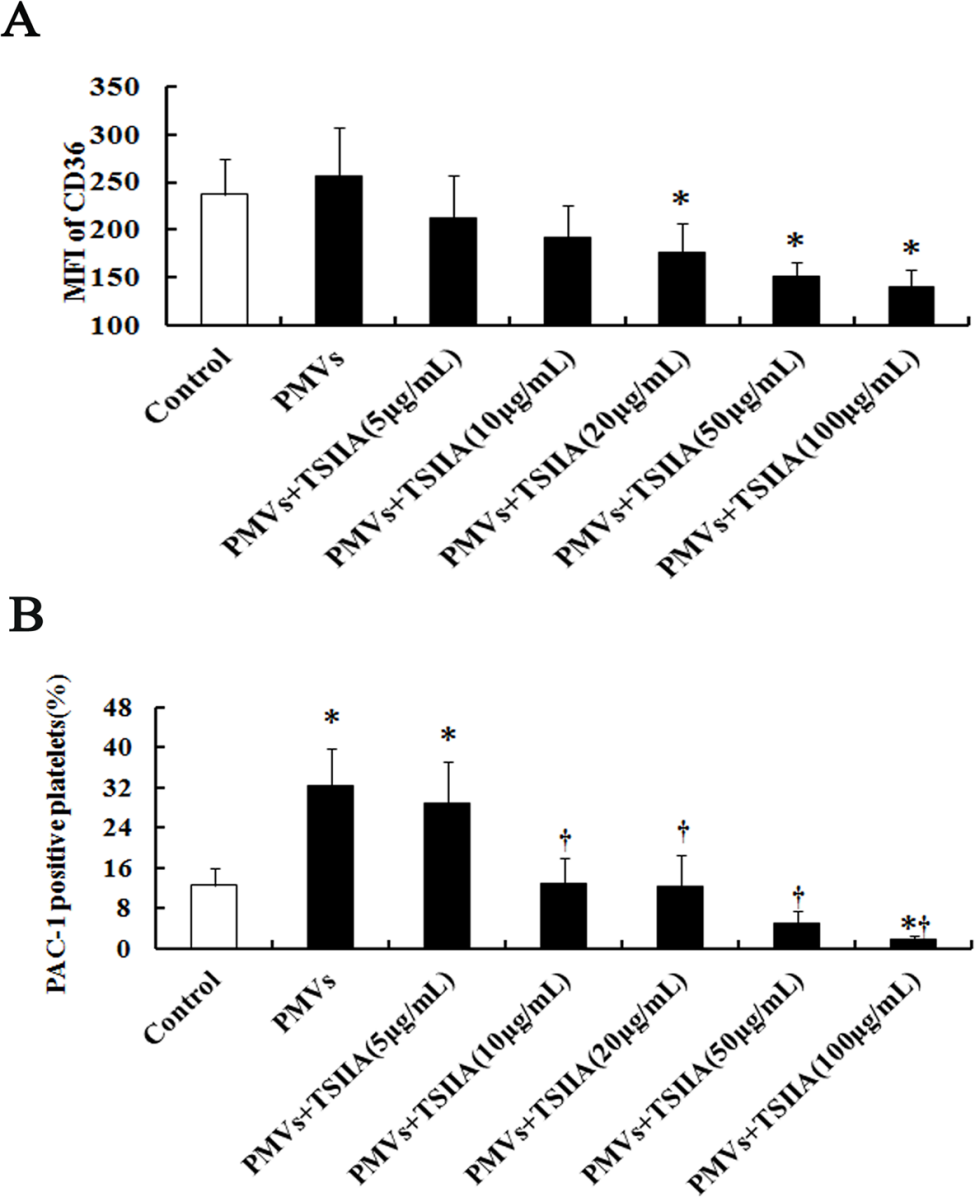
Effect of TanshinoneIIA (TS IIA) on platelet CD36 level and platelet activation. Quantification of flow cytometry of platelets incubated with serial concentrations of TS IIA for 15 min before stimulation with 10 μg/mL PMVs, then CD36 MFI **a** and PAC-1 positivity **b** was measured. Data are means ± SD (*n* = 4). * *P* < 0.05 compared with untreated control. # *P* < 0.05 compared with PMV treatment

To test the effect of TS IIA on platelet CD36, a receptor of PMVs, we treated resting platelets (1 × 10^6^/mL) with serial doses of TS IIA (5–100 μg/mL) before incubation with PE-conjugated anti-CD36 antibody. The expression of platelet CD36 did not change in response to IL-6–PMVs. TS IIA could prevent the binding of PE-conjugated anti-CD36 antibody to platelets in a concentration-dependent manner. TS IIA with the concentration of 20 μg/mL decreased the expression of CD36 significantly. With the increase of TS IIA concentration, the inhibitory action is stronger (Fig. 3a).

### TS IIA prevents platelet activation

To further examine the effect of TS IIA on platelet CD36 level, we determined the expression of integrin αIIbβ3, a platelet activation marker. IL-6–PMVs significantly enhanced the expression of integrin αIIbβ3, which could be attenuated significantly by 10 μg/mL TS IIA. In addition, TSIIA (5–100 μg/mL) dose-dependently prevented the activation of platelets (Fig. 3b).

### TSIIA downregulates the PMV-activated phosphorylation of MKK4/JNK2

**Fig. 4:**
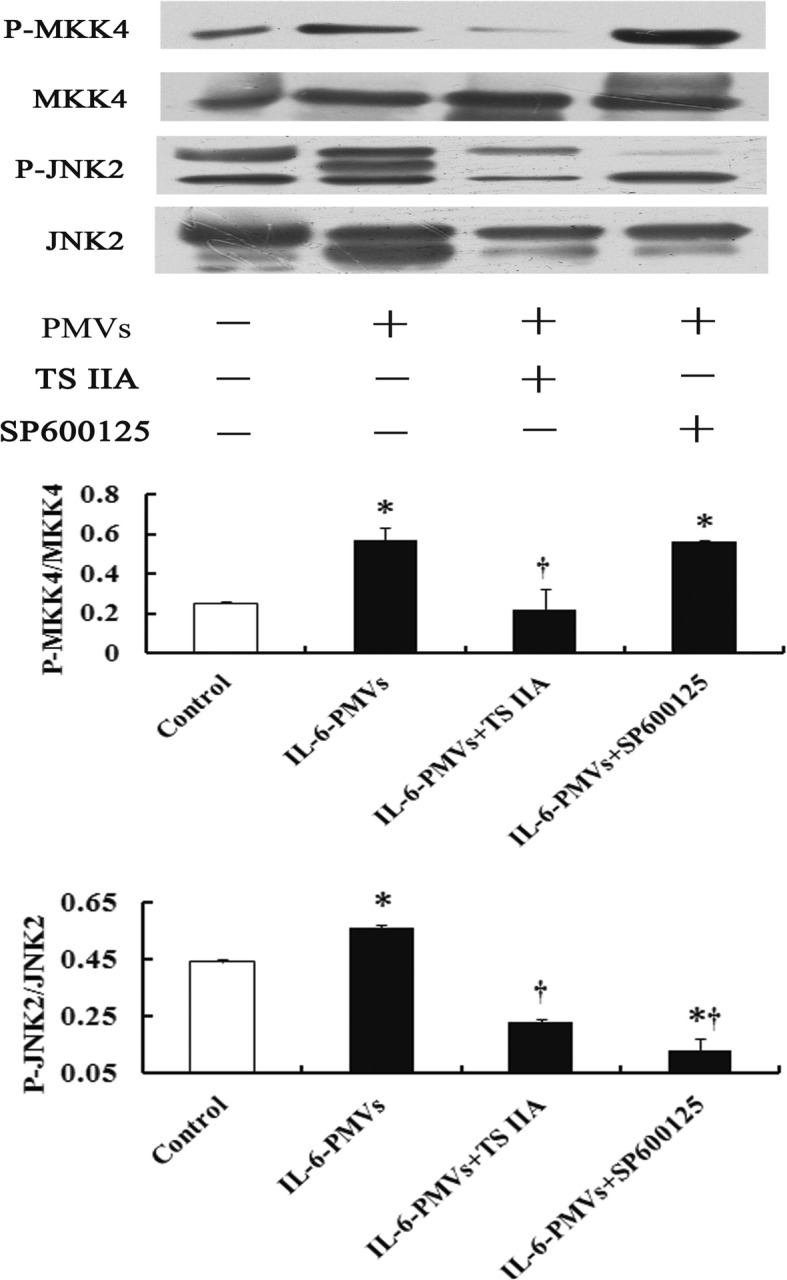
Effect of TS IIA on MKK4/JNK2 pathway. Western blot analysis of phosphorylation of MKK4 and JNK2 after treatment with TS IIA (20 μg/mL) for 15 min in CD36-positive platelets incubated with PMVs. Data are means ± SD (*n* = 3). * *P* < 0.05 compared with untreated control; # *P* < 0.05 compared with PMV treatment

Next, we tested the effect of TS IIA on the MKK4/JNK2 signal pathway by immunoblotting. Resting platelets exposed to IL-6–PMVs for 30 min showed a significant increase in phosphorylation of JNK2 and its upstream activator MKK4. As expected, TS IIA administration lead to downregulation of the phosphorylation of MKK4/JNK2 in platelets pretreated by IL-6–PMVs. The effect of TS IIA was comparable to that of SP600125, a pharmacological inhibitor of JNK (Fig. 4).

## Discussion

Our work showed that TS IIA in vitro inhibits, in a concentration dependent manner, platelet activation induced by PMVs and downregulates CD36 and MKK4/JNK2 signaling pathway. CD36 is speculated to be attractive target of atherosclerosis (AS)-related thrombosis.

TS IIA is a diterpene quinone isolated from dried roots of salvia miltiorrhiza bunge, also known as Danshen, that has traditionally been used in Asian countries for the treatment of atherosclerotic disorders, including coronary artery disease, myocardial infarction and stroke. Thromboembolic events are lethal consequences of AS. More than 150 years ago, Virchow proposed a triad of events needed for thrombus formation—abnormalities of the vessel wall, blood flow, and blood constituents [15]. An update of Virchow’s triad for thrombus formation can be considered as: endothelial dysfunction; abnormal blood stasis; and abnormal platelets, coagulation, and fibrinolysis [16]. Platelets are the central part of the blood constituents, which have long been implicated in the pathogenesis of AS as major components of thrombosis, or as constituents of atheroma [3]. Although there is evidence showing that TS IIA has an anti-platelet effect, its underlying mechanism of anti-platelat effect is not very clear. Here, we investigated the effect of TS IIA on platelet activation and demonstrated that platelet CD36 may be the target of TS IIA.

CD36 is a multifunctional glycoprotein that contributes to several important pathological processes highly relevant to AS. Mice with CD36 deficiency exhibited a greater than 77% decrease in aorta lesions despite the induction of a very high atherogenic milieu [17]. Geloen et al. identified two CD36 inhibitors which were able to rescue well characterized animal models from AS [18]. In the previous study [11], we found that PMVs were ineffective for CD36-deficient platelets. The PMVs derived from oxidized Low Density Lipoprotein (oxLDL)–stimulated platelets could not enhance integrin αIIbβ3 expression in CD36-deficient platelets. The volunteers with CD36 deficiency have not any history of bleeding diathesis. In the present study, we found that TS IIA could decrease the expression of platelet CD36, leading to inhibition of platelet activation.

We have showed that the PMV-CD36 complex activates MKK4/JNK2 signals and contributes to platelet activation [11]. Earlier studies demonstrated that TS IIA can reduce oxidative stress and regulate apoptosis by suppressing JNK and p38 MAPK activation [19]. Nevertheless, the effect of TS IIA on platelet MAPK signal pathways is unknown. Therefore, we explored whether the signal pathway mediated by PMV–CD36 complex could be a potential target for preventing the AS-related thrombosis. Phosphorylation of JNK2 is the end-stage of the pathway; however, inhibition of the MKK/JNK pathway may induce unexpected complications because the activation of the MKK/JNK signal pathway regulates a range of biological processes and occurs inside platelets [20, 21]. The PMV–CD36 complex on the surface of platelets is easier to block. Thus, we focused on targetting the PMV–CD36 complex. TS IIA could block platelet CD36 expression, reduce the production of PMVs induced by IL-6 and inhibit platelet activation. In addition, TS IIA could down-regulates the MKK4/JNK2 phosphorylation. Finally, TS IIA could inhibit the platelet activation stimulated by PMVs. Our results are consistent with several studies showing that TS IIA could inhibit platelet aggregation [22–24]. The PMV–CD36 complex may be a target for preventing the AS-related thrombosis. Of course, there are many other proteins that have influence on the MKK4/JNK2 signaling pathway. This study mainly focus on the PMV-CD36 complex, others also may need to be studied in future.

## Conclusion

In conclusion, our results indicated that TS IIA could inhibit platelet activation induced by PMVs and down-regulates CD36 and MKK4/JNK2 signaling pathway. This study may provide a new strategy for the treatment of AS-related thrombosis by targeting platelet CD36.

## Data Availability

The datasets used and/or analysed during the current study available from the corresponding author on reasonable request.

## Abbreviations

ADP: Adenosine diphosphate
AS: Atherosclerosis
BSA: Bovin serum albumin
FITC: Fluorescein isothiocyanate
IL-6: Interleukin 6
IL-6–PMVs: Microvesicles derived from IL-6 stimulated platelets
JNK2: Jun N-terminal kinase 2
MKK4: Mitogen-activated protein kinase kinase 4
oxLDL: Oxidized low density lipoprotein
PE: Phycoerythrin
p-JNK: Phospho-JNK
p-MKK4: Phospho-MKK4
PMVs: Platelet-derived microvesicles
PRP: Platelet-rich plasma
TS IIA: Tanshinone IIA
